# Supplementary material for: Associated risk factors of severe dengue in Reunion Island: A prospective cohort study
Source: PLoS Negl Trop Dis. 2023 Apr 17;17(4):e0011260. doi: 10.1371/journal.pntd.0011260 (PMC10138848; doi:10.1371/journal.pntd.0011260)
Supplement: S1 Table — Legend: SBP: systolic blood pressure, DBP: diastolic blood pressure, SpO2: saturation pulse oxygen, ECG: electrocardiography, KDIGO: Kidney Disease Improving Global Outcomes, AST: aspartate-amino-transferase, ALT: alanine-amino-transferase (DOCX) [file pntd.0011260.s001.docx]

**S1 Table: Definitions of organ failure according to learned societies [1-5].**

| **Failure** | **Definition** |
| --- | --- |
| Shock [1, 5] | Plasma leakage associated with arterial hypotension or clinical signs of peripheral circulatory failure  Arterial hypotension was defined as systolic blood pressure (SBP) < 90mmHg or mean arterial pressure < 65mmHg (calculated with the following equation (SBP + 2x Diastolic Blood Pressure (DBP)) / 3)  Peripheral circulatory insufficiency was defined by skin mottling, increased skin recoloration time > 3 seconds, cold or cyanotic extremities |
| Respiratory failure [1] | Plasma leakage associated with fluid accumulation with acute respiratory distress  Acute respiratory distress was defined by a SpO2 < 95% and a respiratory rate > 24 / min or the use of oxygen therapy or ventilatory assistance |
| Heart failure [2] | Chest pain, an ECG compatible with the diagnosis of myocarditis and/or pericarditis analyzed by an experienced anesthetist-resuscitator or troponinemia > 14 ng/L, and a transthoracic ultrasound finding impaired left ventricular function |
| Neurological failure [3] | A deterioration in the state of consciousness rated by a Glasgow score <= 13 |
| Renal failure [4] | KDIGO Stage 3, defined by serum creatinine ≥ baseline serum creatinine x3, when this was not available, the higher plasma creatinine was used (≥ 354 mM), recourse to extra-renal purification or diuresis < 0.3mL/kg/h > 24h or anuria > 12h |
| Liver failure [1] | AST and/or ALT ≥ 1000 IU/L |
| Severe bleeding [1] | Defined by cerebro-meningeal hemorrhage, intra-alveolar hemorrhage, hemoptysis, rectal bleeding, hematemesis or transfusion of labile blood product |

SBP : systolic blood pressure, DBP : diastolic blood pressure, SpO2 : saturation pulse oxygen, ECG : electrocardiography, KDIGO : Kidney Disease Improving Global Outcomes, AST : aspartate-amino-transferase, ALT : alanine-amino-transferase

**References**

1. World Health Organization. Dengue guidelines for diagnosis, treatment, prevention and control : new edition. World Health Organization; 2009. Report No.: WHO/HTM/NTD/DEN/2009.1. Available: https://apps.who.int/iris/handle/10665/44188

2. Hékimian G, Franchineau G, Bréchot N, Schmidt M, Nieszkowska A, Besset S, et al. Diagnostic et prise en charge des myocardites. Médecine Intensive Réanimation. 2017 [cited 13 May 2022]. doi:10.1007/s13546-017-1273-4

3. Stahl JP, Azouvi P, Bruneel F, De Broucker T, Duval X, Fantin B, et al. Guidelines on the management of infectious encephalitis in adults. Med Mal Infect. 2017;47: 179–194. doi:10.1016/j.medmal.2017.01.005

4. Cherni N, Jamoussi A, Merhebène T, Ayed S, Ben Khelil J, Besbes M. RIFLE, AKIN et KDIGO en réanimation : quelle classification pour l’insuffisance rénale aiguë au cours du choc septique ? Néphrologie Thérapeutique. 2019;15: 369–370. doi:10.1016/j.nephro.2019.07.259

5. World Health Organization. Dengue haemorrhagic fever : diagnosis, treatment, prevention and control. World Health Organization; 1997. Available: https://apps.who.int/iris/handle/10665/41988
