# Supplementary material for: Associated risk factors of severe dengue in Reunion Island: A prospective cohort study
Source: PLoS Negl Trop Dis. 2023 Apr 17;17(4):e0011260. doi: 10.1371/journal.pntd.0011260 (PMC10138848; doi:10.1371/journal.pntd.0011260)
Supplement: Attachment — Submitted filename: Review Comments_Nov2022.docx [file pntd.0011260.s003.docx]

This study has an important message useful for the clinical management of dengue patients. However, there is a strong need for major revisions of the paper before it can be accepted for publications.

Abstract

In the conclusion, the authors claim that SD is a frequent cause of hospitalization. However, nowhere in the findings had authors reported on this. Authors are suggested to revise this statement.

Introduction

Line 64-65. The incidence as per the reference is reported at ~104 million, whereas authors have cited it as 110 million per year. This needs to be corrected as it is a gross error from the reference.

Line 68. Suggested to replace numeric “4” with the text “four”

Line 68. Suggested to remove “disease” for consistency purpose. In all the above section, authors used only “dengue” and not as “dengue disease”

Line 78-79. Why is organ failure specifically mentioned here? Should’nt it be included in the SD? Its not clear

Methods

Line 85-88. It is not clear what the authors are trying to say in the first paragraph. This paragraph also does not have a heading.

Line 89-92. This whole paragraph is not clear, probably related to the language and sentence structure. For example, the sentence “Inclusion criteria were to have a positive blood sample for DENV by RT-PCR” is understood as recruiting blood samples rather than patients. It might be better read as “patients positive for DENV by RT-PCR were included…..”

Line 95-97. Readers want to know what tools or methods were used to collect data during the follow-up period. Did the authors involve a phone call or were they summoned physically to the study site?

Line 99. Suggested to explicitly describe what biological data were collected.

The authors have not mentioned the variables included in the analysis and how they were collected, measured, or something like this.

Line 101-102. Suggested to relocate this sentence (A bivariate anlysis……defined by the WHO in 2009) just before the multivariate analysis to for ease of understanding.

Line 102-106. It is important to clarify the conditions as to why t test or Mann Whitney test, and, Chisquare or exact test were used. In other words, when was t-test used, when was Mann whitney test used? You might say like this, t test was used in normally distributed data, while non-normal data were tested using Mann Whitney test, something like this.

Line 106. Please remove period(.) after the percentages.

Line 108-110. It is difficult to understand the sentence beginning with “The factors…..in the final model”. The sentence is probably too long, might be clearer if cut short.

The authors have not mentioned how collinearity among variables was assessed. Collinearity among variables can potentially impact the magnitude of the association.

I am surprised that authors have not used the “follow up” data in the analysis. It would be interesting to look at how the various follow-up periods impact the risk of SD.

Since the authors adopted a cohort study design, I am wondering if the odds ratio is the right measure of association. This is only my opinion and not necessary to make changes if deemed redundant.

Line 122. Why were some patients excluded from the analysis? Only 163 were included from the 172 eligible patients.

Line 124-125. Check the sentence structure please

Line 125-126. What is meant by “that year” in the sentence, the most common serotype identified that year was DENV-2.

Line 156. As alluded before, since this is a cohort study, you cannot measure prevalence. It has to be either incidence proportion or incidence density. Or authors might have to change the study design.

Line 158. How do you define kidney injury? This has to be clearly stated in the method section.

Table 1 should capture the test statistic in the corresponding p values, whether it is t test or Chisquare tes?

Line 174-177. I am surprised authors chose to place the main finding in the supplementary file. It should be in the main text.

Line 181. What does “several” mean?

Line 182-183. Have you ever mentioned DENV serotypes in the result section?

Line 210. The younger population has fragile capillaries, which is clearly stated in the literature, as the most common reason of SD. You might want to add this and provide credible reference.

Authors should discuss on this, time elapsed between illness onset and presentation to hospital, as it is statistically significant.
