# Supplementary material for: Associated risk factors of severe dengue in Reunion Island: A prospective cohort study
Source: PLoS Negl Trop Dis. 2023 Apr 17;17(4):e0011260. doi: 10.1371/journal.pntd.0011260 (PMC10138848; doi:10.1371/journal.pntd.0011260)
Supplement: Attachment — Submitted filename: Response to reviewers.docx [file pntd.0011260.s004.docx]

Dear Drs Mellisa Roskosky, Elvina Viennet and Reviewers,

Please find attached our revised manuscript (PNTD-D-22-01199), entitled ‘Associated risk factors of severe dengue in Reunion Island: A prospective cohort study’, by Mathys Carras, Olivier Maillard, Julien Cousty, Patrick Gérardin, Malik Boukerrou, Loïc Raffray, Patrick Mavingui, Patrice Poubeau, André Cabie, and Antoine Bertolotti, which we are resubmitting for publication as an Original Article in *PLOS Neglected Tropical Diseases.*

We want to sincerely thank all of you for your useful remarks that have helped us improve our paper and clarify its message. We took into consideration all your comments in revising our manuscript.

Our responses to the comments are detailed below. Comments are in blue, responses are in black and recommended changes are in red in the text and in the manuscript.

**REVIEWER 1**

We wish to thank the reviewer for his very helpful comments

Comment: Please clarify in the text what symptom/s were used for patient recruitment.

Response: We have added in the manuscript (page 5, lines 97-100): “They [The patients] must have presented at least two symptoms among the following: fever (documented or declared), headache, rash, myalgia, arthralgia, abdominal pain or tenderness, bleedings and/or low platelets blood level below 150 G/l. Children younger than 6 years of age need to present fever (documented or declared by the family) with or without pain on an age-adapted visual analogue scale.”

We also have modified the beginning of the study population section (lines 93-95): “~~Patient inclusion was conducted on a subset of Reunion Island patients~~ All patients suspected of dengue fever who were admitted to emergency departments of the University Hospital of Reunion from 1st of January to 30th of June, 2019, were eligible.”

Comment: What percentage of patients met the case definition of severe dengue at the time of recruitment?

Response: Twenty four patients (15% of total, 65% of SD) were classed as severe at the time of recruitment (based on clinical presentation and biology or other exams run at this time). We then have removed the heading of the paragraph (page 8, line 179): “~~Prevalence of severe dengue during the acute phase~~” and have modified the beginning of the paragraph (page 8, line 180-183): “Of the 163 patients included, 37 (23%) were classed as severe of whom 24 (65%) at the time of recruitment,~~presented with SD, including~~ 14 (38%) with shock, 14 (38%) with severe haemorrhage, 23 (62%) with severe organ failure dominated by kidney failure ~~injury~~ (n=9) and 1 (3%) which resulted in death.”

Comment: Confusion is a subjective factor and therefore cannot be used for prediction. Authors seem to indicate in the abstract and conclusion that identified factors can predict who will develop severe dengue disease but in realty these are risk factors that are associated with severity and may or may not accurately predict severity of a disease. For example, diabetes, hypertension and various other factors correlated with severe dengue in various other dengue studies but did not associate in this cohort.

Response: We agree with the reviewer and have modified the abstract accordingly (page 2, lines 28-29): “However, there is no consensus in the literature on factors ~~which can predict~~ associated with severity of illness.”. We also clarified the conclusion as follows (page 16, line 276-279): “Our results will be used in other large-scale studies during future dengue epidemics in Reunion Island, which ~~They~~ will allow the identification of predictive factors in the evolution towards a more severe form of the disease, as well as the drafting of referral protocols to specialized services adapted to the profile of the patient.”

Comment: Line 108 authors meant p values of < 0.02 or 0.2? Age was not significantly associated in the bivariate analysis and so why was it included in the multivariate analysis?

Response: Variables with P value below 0.2 were introduced in the multivariate regression, and not only significant variables identified in the univariate analysis. It is usually made using this threshold to look for potential confounding variables which could be over 0.05 in univariate analysis.

Indeed, age was not associated with severity in the univariate analysis with a P>0.2. Thus it had not been introduced into the multivariate model, but was forced in the final model, as is often the case for age and gender. However, the significancy did not make sense in our study when looking at the results of univariate analysis, and a stratified analysis for age is poor and inconclusive due to sample size. To avoid misunderstanding, age and gender were withdrawn from the multivariate final model. We have modified accordingly the abstract (page 2, lines 38-42): “The factors ~~identified~~ ~~in multivariate analysis~~ associated with SD in multivariate analysis were ~~age (odds ratio [OR]: 0.97, 95% confidence interval [CI]: 0.94-1.00),~~ a history of cardiovascular disease (OR: 2.67, 95%CI: 1.13-6.29), Western European origin (OR: 18.44, 95%CI: 2.00-170) and the time between the first symptom and hospital consultation (OR: 1.20, 95%CI: 1.03-1.40)”, and the end of the result section (page 13, lines 200-203): “Multivariate analysis identified an increased risk of SD in ~~children (OR: 0.97, 95%CI: 0.94-1.00),~~ patients with a history of cardiovascular disease (OR: 2.67, 95%CI: 1.13-6.29), Western European origin (OR: 18.44, CI: 2.00 - 170), and time from first symptom to consultation (OR: 1.20, CI: 1.03 - 1.40) (Table 2).”

Furthermore, as requested, the table was moved from the supporting information file to the manuscript (page 13, line 205-209). The main difference with the previous final model is a decrease of cardiovascular disease OR, which is not adjusted for age anymore. But the global message addressed by multivariate analysis remains the same.

**Table 2: Factors associated with severity of dengue in adults with positive ~~Dengue~~ RT-PCR at disease onset. Multivariate analysis using logistic regression. CARBO cohort study, Réunion Island 2019 (N = 163).**

| **Variables** | **Adjusted Odds Ratio (95%CI)** | **P-value** |
| --- | --- | --- |
| Time elapsed between illness onset and presentation to hospital | 1.20 (1.03 - 1.40) | 0.021 |
| West European origin | 18.44 (2.00 - 170) | 0.010 |
| Cardiovascular disease | 2.67 (1.13 – 6.29) | 0.025 |

95%CI: 95% confidence interval

Comment: Viral titers or ns1 levels will be important to report in this dataset.

Response: Viral loads and NS1 levels have not been documented in the study as RT-PCR was qualitative and no NS1 ELISA were made. Only qualitative assessment of NS1 Antigen by RDT was done.

RDT was the SD Bioline Dengue Duo (Standard Diagnostics). This is an immunochromatographic one-step assay to detect simultaneously the NS1 antigen and IgM and IgG DENV antibodies.

We have added information on RT-PCR used for diagnosis in the method section (page 5, lines 101-105): “The Tropical Fever Core multiplex RT-PCR (Fast Track Diagnostics) was used for on-site diagnosis. It was a conventional two step real-time RT-PCR, which could detect dengue (without differentiation between serotypes), chikungunya and West Nile viruses, *Leptospira spp.*, *Rickettsia spp.* and *Salmonella spp.*, and *Plasmodium spp.,* which are pathogens that could circulate in the South West Indian Ocean region.”

Comment: Please describe which co-infections were reported.

Response: Of 19 (12%) co-infected patients, 9 urinary tract infections, 5 bacteriemia, 4 pulmonary infections, 3 gastroenteritis and 1 cutaneous infection, were reported. Fifteen (79%) were diagnosed at admission. Five patients had complications related to their coinfection.

As coinfections were not associated with severity, no more information was added in the manuscript.

Comment: Dengue reinfection- how it’s determined and whether it was homologous or heterologous reinfection? Please describe in the text.

Response: Dengue reinfection was not systematically documented in 2019 because Reunionese people were considered naïve to dengue at this time, and serum neutralization was not routinely done.

Comment: Multivariate- what set of independent variables were used in multivariate analysis? Full table should be reported in the supplemental file.

Response: It is unusual to present the initial multivariate model, but it is explained in the method section that all variables with *p*-value below 0.2 in univariate analysis were introduced.

Comment: For some factors such as age less than 18years and Western European the sample size is too small to make any meaningful determination.

Response: We agree with the reviewer concerning age and it was removed from the multivariate final model (along with gender, which was also forced into the final model). For Western European origin, even if the 95% confidence interval (CI) is large, OR is high enough to be detected with confidence with such a sample size. But as mentioned in the discussion, these findings need to be consolidated by further studies.

Our sample size is able to detect at least OR ranging from 2.8 to 4.4, depending on the proportion of the characteristics in the groups (from PASS 2020. NCSS, LLC. Kaysville, Utah, USA). Indeed, we lack the power to detect the bottom limit of the 95%CI of OR for Western European origin, but we are confident with at least an 80%CI (4.3-79). If we randomly draw a new sample from the same population of dengue fever, we would have an 80% chance of finding an OR in this interval.

**REVIEWER 2**

We wish to thank the reviewer for his very helpful comments

Comment: The objectives are clearly stated and hypothesis covered the factors included by the WHO classification. The design is adequate.
The population description requires more details for those who are not local to Reunion Island, the issue is what they cover in the classification of ethnic groups and how other groups are classified (maybe they include all in other?).
What is the definition of Indian and Asian ethnicities please specify (line 98 , page 10)

Response: Ethnic groups were defined as follows in the study protocol:

Parent or ancestor from Sub-Saharan Africa, India, Western Europe, South-east Asia; Native American; or other. When ‘other’, it should be specified, but there are various responses due to the multi-ethnic population of Reunion.

We have modified the text accordingly (page 5, lines 112-115) : “Ethnicity was identified by patient self-determination ~~six categories were available: African, Indian, Asian, Western European, Native American, or other~~ and divided into six categories: existence of a parent or an ancestor from Sub-Saharan Africa, India, West Europe, South-east Asia; being Native American; or from other origin(s).”

Comment: The sample size is adequate for most analysis.This number of cases for those younger than 18 is relatively small. This can limit the interpretation. The description of the statistical analysis seems correct but lacks some details specially in the clear flow in the use of specific tests
No concerns about ethical  or regulatory requirements.

Response: As age was removed from the multivariate final model (please see response to the next comment), it is less relevant to discuss, but we agree with the reviewer it should have been addressed.

The data analyses section and its heading were revised (page 5-6, lines 119-135):

“**Data Analyses ~~and Statistical Tests~~**

Quantitative variables were described with means and standard deviations, while categorical variables were described as numbers and percentages.

A bivariate analysis was performed to compare clinical and laboratory features of DF patients according to severity of illness (defined by the WHO in 2009). Gaussian variables were compared using the Student’s t-test, while non parametric continuous variables were compared using the Wilcoxon Mann Whitney test. The normality of the distributions was checked by the Shapiro-Wilk test, and the homogeneity of variances by the Levene test. Categorical variables were compared using Chi-square test or the Fisher’s exact test, as appropriate.

A multivariate logistic regression was performed to identify factors associated with SD. Variables with a *p*-value <0.2 in bivariate analysis were entered into a multivariate model. In the case of collinearity detected by using the variance inflation factor (VIF), the most relevant variable was retained in the model. A backward stepwise automated procedure was used to select factors associated with *p*-value <0.05 in the final model. Adjusted odds ratios (OR) and their 95% confidence intervals (95%CI) were derived from the regression coefficients. Data were analyzed using SPSS software (IBM SPSS 23.0, IBM Corp. Armonk, NY, USA). All tests were two-tailed and the significance level was set at 0.05. “.

Comment: The results seem according to the analysis plan however there are some issues with the interpretation of the multivariate analysis, the ORs and CI for the variable Age provide the idea of a protective effect in general, but affirming that population under 18 years is at high risk requires to show the analysis of the different age groups and ORs and CI greater than 1 (maybe they just need to do it more explicit in the manuscript). The multivariate analysis table should be in the main text just like the descriptive statistics

Response: As age was not associated with severity in the univariate analysis with a *p*-value >0.2, it had not been entered into the multivariate model, but was forced at the end into the final model, as age and sex usually are. However, the significancy did not make sense in our study when looking at the results of univariate analysis, and a stratified analysis for age is poor and inconclusive due to sample size. To avoid misunderstanding, age and gender were withdrawn from the multivariate final model. Furthermore, as requested, the table was moved from the supporting information file to the manuscript (page 13, line 205-209).

**Table 2: Factors associated with severity of dengue in adults with positive ~~Dengue~~ RT-PCR at disease onset. Multivariate analysis using logistic regression. CARBO cohort study, Réunion Island 2019 (N = 163).**

| **Variables** | **Adjusted Odds Ratio (95%CI)** | **P-value** |
| --- | --- | --- |
| Time elapsed between illness onset and presentation to hospital | 1.20 (1.03 - 1.40) | 0.021 |
| West European origin | 18.44 (2.00 - 170) | 0.010 |
| Cardiovascular disease | 2.67 (1.13 – 6.29) | 0.025 |

95%CI: 95% confidence interval

Comment: Provide more information about the breakdown of the population in ethnic groups, and clarify the process of identification of Age (and the category < 18 yrs as risk factor, State with clarity if the data support the serotype 2 as responsible for the described epidemic.

Response: We have clarified above the definition of ethnic group classifications, and removed age (and gender) from the multivariate final model.

The DENV-2 serotype has been mostly identified over the study period, as mentioned in the discussion (page 14, lines 214-215), but it was on a sample of patients, as the RT-PCR kit did not allow dengue differentiation, and serotype was not routinely determined.

Comment: My general impression is that this study was well designed and conducted considering the clinical setting and the unpredictable flow of cases in an epidemic, however the manuscript needs to be expanded to explain the process of handling the variables in the multivariate analysis compared with the univariate analysis

Response: We agree with the reviewer and have modified the statistical analysis section accordingly.

**REVIEWER 3**

We wish to thank the reviewer for his very helpful comments

Comment: There is a need to revamp method section. It is not clear on the type of study design and measure used to assess difference between severe and non-severe dengue. Study population is not clearly defined and sample size was not reported.

Response: As a cohort could be retrospective or historical, we have modified as follows for a better comprehension of the study design (page 4, line 90-91): “~~The cohort, named~~ CARBO (Cohorte ~~des~~ ARBOviroses) is a French multicentric prospective cohort study dedicated to the understanding of the acute stage of arboviral infections.”

We also added this sentence (page 5, lines 107-108): “The primary outcome was the determinants associated with severity by comparing characteristics of SD patients with non-SD patients”

To improve the description of the study population, the paragraph was modified as follows (page 4-5, lines 93-100): “~~Patient inclusion was conducted on a subset of Reunion Island patients~~ All patients suspected of dengue fever who were admitted to emergency departments of the University Hospital of Reunion from 1st of January to 30th of June, 2019, were eligible. Patients were recruited if they had a positive blood sample for DENV by RT-PCR, and onset of symptoms ≤7 days (until 21 days for SD). They must have presented at least two symptoms among the following: fever (documented or declared), headache, rash, myalgia, arthralgia, abdominal pain or tenderness, bleedings and/or low platelets blood level below 150 G/l. Children younger than 6 years of age need to present fever (documented or declared by the family) with or without pain on an age-adapted visual analogue scale.”

The study sample was composed of all the recruited patients during the 2019 epidemic in Reunion. Thus, the sample size was derived from the number of patients recruited over the epidemic period. It is able to detect at least OR ranging from 2.8 to 4.4, depending on the proportion of the characteristics in the groups (from PASS 2020. NCSS, LLC. Kaysville, Utah, USA)

The method section was revised and recruiting criteria more clearly presented.

Comment: The main finding in placed in the supplementary file. It has to be relocated in the main content.

Response: We agree with the reviewer, and the table showing results of the multivariate analysis was removed in the main text, with some modifications required by the other reviewers (age and gender were withdrawn from the table as they were not retained by the regression, but forced into the multivariate final model) (page 13, line 205-209).

**Table 2: Factors associated with severity of dengue in adults with positive ~~Dengue~~ RT-PCR at disease onset. Multivariate analysis using logistic regression. CARBO cohort study, Réunion Island 2019 (N = 163).**

| **Variables** | **Adjusted Odds Ratio (95%CI)** | **P-value** |
| --- | --- | --- |
| Time elapsed between illness onset and presentation to hospital | 1.20 (1.03 - 1.40) | 0.021 |
| West European origin | 18.44 (2.00 - 170) | 0.010 |
| Cardiovascular disease | 2.67 (1.13 – 6.29) | 0.025 |

95%CI: 95% confidence interval

**REVIEWER ? (from the file attached)**

We wish to thank the reviewer for his very helpful comments

Comment: Abstract

In the conclusion, the authors claim that SD is a frequent cause of hospitalization. However, nowhere in the findings had authors reported on this. Authors are suggested to revise this statement.

Response: In table 1 (page 10) it is presented that 32 (87%) DS patients were hospitalized vs 91 (72%) non DS patients, *p*=0.063. Moreover DS patients stayed longer than non-DS patients which is statistically significant (*p*=0.013)

Comment: Introduction

Line 64-65. The incidence as per the reference is reported at ~104 million, whereas authors have cited it as 110 million per year. This needs to be corrected as it is a gross error from the reference.

Response: We agree with the reviewer and have corrected to match with the reference, even if presented with a large confidence interval (page 3, lines 64-65): “Dengue is the most common mosquito-borne arbovirosis. In 2017, its incidence was ~~measured~~ estimated at more than 104 million people, of which 40,000 induced a death [1,2].”

Comment: Line 68. Suggested to replace numeric “4” with the text “four”

Response: It was modified in the text (page 3, line 67-68): “Dengue is caused by four viral serotypes (DENV 1-4) with significant genotypic variability”

Comment: Line 68. Suggested to remove “disease” for consistency purpose. In all the above section, authors used only “dengue” and not as “dengue disease”

Response: It was modified accordingly in the text (page 3, line 67-69): “Dengue is caused by four viral serotypes (DENV 1-4) with significant genotypic variability~~.~~ ~~Dengue disease~~ and is characterised by a large clinical polymorphism with numerous signs of varying severity.”

Comment: Line 78-79. Why is organ failure specifically mentioned here? Should’nt it be included in the SD? Its not clear

Response: Organ failure is included in the definition of SD (WHO, 2009) but it was lacking accuracy, therefore prognostic factors resulting from analyses vary, depending on whether definitions of organ failure are retained. We have added to the end of the sentence (page 4, line 80): “The performance of prognostic factors for SD varies according to the studies and the definitions of organ failure used when defining SD”

Comment: Methods

Line 85-88. It is not clear what the authors are trying to say in the first paragraph. This paragraph also does not have a heading.

Response: We agree with the reviewer. The study protocol was proposed in 2009 by the University Hospital of Martinique which was experiencing a dengue outbreak. To gain time when facing our first epidemic, this protocol was chosen to be used for studying dengue in Reunion rather than proposing another protocol.

The paragraph was improved for better comprehension and included in the first heading of the method section i.e. “Study population” (page 4, line 90-93): “~~The cohort, named~~ CARBO (Cohorte ~~des~~ ARBOviroses) is a French multicentric prospective cohort study dedicated to the understanding of the acute stage of arboviral infections. It was proposed ~~in collaboration with~~ by the university hospital of Martinique and is registered on clinicaltrials.gov (NCT01099852).”

Comment: Line 89-92. This whole paragraph is not clear, probably related to the language and sentence structure. For example, the sentence “Inclusion criteria were to have a positive blood sample for DENV by RT-PCR” is understood as recruiting blood samples rather than patients. It might be better read as “patients positive for DENV by RT-PCR were included…..”

Response: We agree with the reviewer and the paragraph was modified accordingly (page 4-5, lines 95-97): “Patients were recruited if they had a positive blood sample for DENV by RT-PCR, and onset of symptoms ≤7 days (until 21 days for SD). “

Comment: Line 95-97. Readers want to know what tools or methods were used to collect data during the follow-up period. Did the authors involve a phone call or were they summoned physically to the study site?

Response: The sentence was modified as follows (page 5, lines 112-114): “Sociodemographic and clinical data were collected by a clinician at each physical exam of the patient via a standardized questionnaire at Day 0 (D0), D3, D5-7, D8-10, D21, D45, D90, D180.”

Comment: Line 99. Suggested to explicitly describe what biological data were collected.

The authors have not mentioned the variables included in the analysis and how they were collected, measured, or something like this.

Response: To avoid redundancy of a listing of the collected data, we propose adding the following sentences at the end of the data collection paragraph (page 5, line 116-118): “All biological exams were carried out in the laboratories of the University Hospital of Reunion. All collected data were reported in a case report form and are detailed in table 1.”

Comment: Line 101-102. Suggested to relocate this sentence (A bivariate anlysis……defined by the WHO in 2009) just before the multivariate analysis to for ease of understanding.

Response: The data analyses section was revamped and this sentence put closer to the multivariate analysis paragraph (page 6, line 122-123)

Comment: Line 102-106. It is important to clarify the conditions as to why t test or Mann Whitney test, and, Chisquare or exact test were used. In other words, when was t-test used, when was Mann whitney test used? You might say like this, t test was used in normally distributed data, while non-normal data were tested using Mann Whitney test, something like this.

Response: We understand the comments of the reviewer but it is unusual to detail all the conditions of application of these common statistical tests. For example normality is not the only condition of applicability of t-test. We already mentioned the tests used to check these conditions. Conversely, Fisher’s exact test should always be used; Chi-square test was developed because of the limits in calculation of computers but there are no limits at all today. However, we keep on using Chi-square if cell counts are higher than 5 in contingency tables.

We have modified the data analyses section as detailed below.

Comment: Line 106. Please remove period(.) after the percentages.

Response: We changed square brackets to parentheses

Comment: Line 108-110. It is difficult to understand the sentence beginning with “The factors…..in the final model”. The sentence is probably too long, might be clearer if cut short.

The authors have not mentioned how collinearity among variables was assessed. Collinearity among variables can potentially impact the magnitude of the association.

I am surprised that authors have not used the “follow up” data in the analysis. It would be interesting to look at how the various follow-up periods impact the risk of SD.

Since the authors adopted a cohort study design, I am wondering if the odds ratio is the right measure of association. This is only my opinion and not necessary to make changes if deemed redundant.

Response: The data analyses section was revamped and its heading modified (page 5-6, lines 119-135):

“**Data Analyses ~~and Statistical Tests~~**

Quantitative variables were described with means and standard deviations while categorical variables were described as numbers and percentages.

A bivariate analysis was performed to compare clinical and laboratory features of DF patients according to severity of illness (defined by the WHO in 2009). Gaussian variables were compared using the Student’s t-test while non parametric continuous variables were compared using the Wilcoxon Mann Whitney test. The normality of the distributions was checked by the Shapiro-Wilk test, and the homogeneity of variances by the Levene test. Categorical variables were compared using Chi-square test or the Fisher’s exact test, as appropriate.

A multivariate logistic regression was performed to identify factors associated with SD. Variables with a *p*-value <0.2 in bivariate analysis were entered into a multivariate model. In the case of collinearity, detected by using the variance inflation factor (VIF), the most relevant variable was retained in the model. A backward stepwise automated procedure was used to select factors associated with *p*-value <0.05 in the final model. Adjusted odds ratios (OR) and their 95% confidence intervals (95%CI) were derived from the regression coefficients. Data were analysed using SPSS software (IBM SPSS 23.0, IBM Corp. Armonk, NY, USA). All tests were two-tailed and the significance level was set at 0.05.”

As 24 (15% of total, 65% of all SD) patients were severe at the time of recruitment (based on clinical presentation and biology or other exams run at this time), and 32 non-SD patients at the time of inclusion (20% of total, 25% of non-SD) were lost to follow-up, a stratified analysis by follow-up periods to study the impact of follow-up on severity is impossible. But it is of interest for further studies. This study was observational and hence there was no standard of management which could bias this type of analysis. The main concern was to identify determinants which could impact a decision to hospitalize patients regarding their features at presentation.

As requested by another reviewer, we have modified the beginning of the paragraph (page 8, line 180-183): “Of the 163 patients included, 37 (23%) were classed as severe of whom 24 (65%) at the time of recruitment,~~presented with SD, including~~ 14 (38%) with shock, 14 (38%) with severe haemorrhage, 23 (62%) with severe organ failure dominated by kidney failure ~~injury~~ (n=9) and 1 (3%) which resulted in death.”

The purpose of CARBO was to constitute a cohort of patients, but the main objective was to identify determinants of severity, which could be done by defining which cases were severe, and by comparing their features before or when they were diagnosed as severe. Thus it is a retrospective analysis and the reason why we used odds ratios (OR) to report association.

Comment: Line 122. Why were some patients excluded from the analysis? Only 163 were included from the 172 eligible patients.

Response: We agree with the reviewer. We have modified Figure 1 by adding a box at the top of the flowchart (Fig 1 file)

Comment: Line 124-125. Check the sentence structure please

Response: The sentence was reworded (page 7, lines 146-149): “Cardiovascular comorbidities were the most ~~common~~ reported medical background (46%), ~~far ahead of~~ followed by hypertension (34%), diabetes mellitus (23%), and obesity (16%) (Table 1).”

Comment: Line 125-126. What is meant by “that year” in the sentence, the most common serotype identified that year was DENV-2.

Response: Yes, this is the meaning but we propose modifying it to be clearer (page 7, line 149): “The most common serotype identified ~~that year~~ in 2019 was DENV-2."

Comment: Line 156. As alluded before, since this is a cohort study, you cannot measure prevalence. It has to be either incidence proportion or incidence density. Or authors might have to change the study design.

Response: We agree with the reviewer. Obviously it is incidence and not prevalence. We have modified the text (page 7, lines 155, 172) and removed the heading of the paragraph (page 7, line 179): “~~Prevalence of severe dengue during the acute phase~~”. Furthermore, we rounded the percentages lines 156-158 (page 7) in accordance with lines 180-184 (page 8).

Comment: Line 158. How do you define kidney injury? This has to be clearly stated in the method section.

Response: We have reworded the sentence (page 8, line 182): “23 (62%) with severe organ failure dominated by kidney failure ~~injury~~ (n=9)”. And renal failure is already defined in the supporting information file (S1 Table, page 2).

Comment: Table 1 should capture the test statistic in the corresponding p values, whether it is t test or Chisquare tes?

Response: We added asterisks in the column P-value and in the legend of table 1 to clarify which tests were used. Incidentally, we noticed an inversion between 2 variables: Preventive and curative anticoagulant. It has been corrected in table 1 (page 9-13).

Comment: Line 174-177. I am surprised authors chose to place the main finding in the supplementary file. It should be in the main text.

Response: We agree with the reviewer and the table showing results of the multivariate analysis was moved to the main text, with some modifications required by the other reviewers (age and gender were withdrawn from the table as they were not retained by the regression, but forced in the multivariate final model) (page 13, line 205-209).

**Table 2: Factors associated with severity of dengue in adults with positive ~~Dengue~~ RT-PCR at disease onset. Multivariate analysis using logistic regression. CARBO cohort study, Réunion Island 2019 (N = 163).**

| **Variables** | **Adjusted Odds Ratio (95%CI)** | **P-value** |
| --- | --- | --- |
| Time elapsed between illness onset and presentation to hospital | 1.20 (1.03 - 1.40) | 0.021 |
| West European origin | 18.44 (2.00 - 170) | 0.010 |
| Cardiovascular disease | 2.67 (1.13 – 6.29) | 0.025 |

95%CI: 95% confidence interval

Comment: Line 181. What does “several” mean?

Response: We agree with the reviewer. As these results are not presented in the result section or in any table, the sentence was modified (page 13-14, lines 212-214): “The warning signs had a low discriminatory value as their proportion was similar between the two groups~~, and only the association of several warning signs showed a tendency in favour of SD~~.”

Comment: Line 182-183. Have you ever mentioned DENV serotypes in the result section?

Response: It was mentioned at the beginning of the result section (Page 7, line 149): “The most common serotype identified in 2019 was DENV-2.”

Comment: Line 210. The younger population has fragile capillaries, which is clearly stated in the literature, as the most common reason of SD. You might want to add this and provide credible reference.

Response: As age was removed from the multivariate final model, it is less relevant to discuss, but we agree with the reviewer it should have been addressed.

Comment: Authors should discuss on this, time elapsed between illness onset and presentation to hospital, as it is statistically significant.

Response: This result is discussed (page15, line 239-240): “A late consultation, which delays intravenous rehydration, could also explain the increased risk of SD.” It could now be more developed as this is no longer a short communication, but the OR is small compared to the other significant features. It was important to adjust for, but its interpretation could be biased as the date of illness onset is declarative.

We hope you will find our revised version acceptable for publication in *PLOS Neglected Tropical Diseases* and look forward to hearing from you.

Yours sincerely,

Dr Olivier MAILLARD, PharmD, MSPH

Dr Antoine BERTOLOTTI, MD, PhD
