# Supplementary material for: Associated risk factors of severe dengue in Reunion Island: A prospective cohort study
Source: PLoS Negl Trop Dis. 2023 Apr 17;17(4):e0011260. doi: 10.1371/journal.pntd.0011260 (PMC10138848; doi:10.1371/journal.pntd.0011260)
Supplement: Attachment — Submitted filename: Response to reviewers.docx [file pntd.0011260.s005.docx]

Dear Drs Mellisa Roskosky, Elvina Viennet and Reviewers,

Please find attached our revised manuscript (PNTD-D-22-01199), entitled ‘Associated risk factors of severe dengue in Reunion Island: A prospective cohort study’, by Mathys Carras, Olivier Maillard, Julien Cousty, Patrick Gérardin, Malik Boukerrou, Loïc Raffray, Patrick Mavingui, Patrice Poubeau, André Cabie, and Antoine Bertolotti, which we are resubmitting for publication as an Original Article in *PLOS Neglected Tropical Diseases.*

We want to sincerely thank all of you for your useful remarks that have helped us improve our paper and clarify its message. We took into consideration all your comments in revising our manuscript.

Our responses to the comments are detailed below. Comments are in blue, responses are in black and recommended changes are in red in the text and in the manuscript.

**EDITORS**

Comment: This version of the manuscript is much improved. There are a few minor revisions that will need to be addressed prior to publication. These revisions include adding some additional detail in the methods section that were provided in your response to reviewers of your original submission and expanding on your conclusions related to between disease onset and hospital presentation. Please see reviewer comments for additional details.

Response: Suggested revisions have been taken into account and the manuscript has been modified accordingly.

**REVIEWER 1**

We wish to thank the reviewer for his helpful comments

Comment: [Comment: Please describe which co-infections were reported.

Response: Of 19 (12%) co-infected patients, 9 urinary tract infections, 5 bacteriemia, 4 pulmonary infections, 3 gastroenteritis and 1 cutaneous infection, were reported. Fifteen (79%) were diagnosed at admission. Five patients had complications related to their coinfection. As coinfections were not associated with severity, no more information was added in the manuscript.]

Please add above response information to the method section since related data is presented in table-1.

Response: The following sentence has been added to the result section rather than the method section (page 7, lines 162-165):” Coinfections were not associated with severity (p=0.146) and out of 19 (12%) co-infected patients, 9 urinary tract infections, 5 bacteremias, 4 pulmonary infections, 3 gastroenteritis and 1 skin infection were reported. They were present at admission in 15 (79%) patients.”

Comment: [Comment: Dengue reinfection- how it’s determined and whether it was homologous or heterologous reinfection? Please describe in the text.

Response: Dengue reinfection was not systematically documented in 2019 because Reunionese people were considered naïve to dengue at this time, and serum neutralization was not routinely done.]

Please add in the method section that DENV re-infection was determined based on the presence of DENV reactive IgG at the time of enrollment using Dengue Duo kit or howsoever authors determined it, if differently.

Response: The following sentence was added to the method section (page 5, lines 100-105):” Serology was not routinely performed, but when there was a strong suspicion of dengue fever and a negative RT-PCR result. It was not systematically double checked three weeks later during the epidemic, or in ambulatory care, so the link between the data could not be made with confidence. For this reason, only cases with positive RT-PCR results were included in the study. Dengue reinfection was not systematically documented in 2019 because Reunionese people were considered naïve to dengue at this time, and serum neutralization was rarely performed.”

**REVIEWER 2**

We wish to thank the reviewer for his helpful comments

Comment: The objectives are clearly stated, and the study design in general is adequate to pursue the mentioned objectives. The description of the population is much clear than before and it contain the elements to test the hypothesis. The sample size goes according to the possibilities to gather a prospective cohort. Statistical analysis is fine and consistent to previous studies, and they support the conclusion. They followed a conventional method to reach their conclusion.

I don't find ethical or regulatory issues to be solved.

Response: No response required

Comment: Analysis was done according to the plan stated by the authors, results are completely presented, Tables reflect the analysis work done by the authors and they convey well the results they obtained. My only concern is how the presentation of results provide enough basis to discuss a richer discussion.

Response: No response required

Comment: The conclusions are supported by the data but they don't expand on the implication of one of the severe dengue predictors:

Time elapsed between illness onset and presentation to hospital. The authors should elaborate what is the practical meaning of that: failure in the early detection of cases, lack of information in the population. The other element to hypothesize is how the cardiovacular co-morbidities interact with that delay.

In general terms the limitations were mentioned. The authors provide the implications of their findings to understand the local characteristics of Severe dengue and how they are consistent to the general epidemiology of dengue.

Even when they approach from a clinical perspective they touch well the Public Health implications.

Response: Submitted initially as a short communication, we had to take into account the word count and therefore tried to present the most relevant findings and discuss them, but we agree with the reviewer that other results could have been discussed.

Time from illness onset to hospital presentation is associated with severity in both univariate and multivariate analyses in our study. This is clear in the univariate analysis but somewhat more difficult to discuss in the multivariate analysis. Basically, one more day elapsed means a 1.2 fold increased risk of developing a severe illness. However onset date is declarative and the time frames are calculated in days, not hours, so a difference of one day is sometimes more or sometimes less. It is more relevant when the delay is categorized, and a delay in consultation greater than 2 days is significantly associated with severity (adjusted OR=2.46, IC95%: 1.42-4.27, p=0.036)

We then proposed the following final multivariate model in the manuscript (page 12, Table 2):

| **Variables** | **Adjusted Odds Ratio (95%CI)** | **P-value** |
| --- | --- | --- |
| Time elapsed between illness onset and presentation to hospital > 2 days | 2.46 (1.42 – 4.27) | 0.036 |
| West European origin | 17.60 (4.15 - 74) | 0.011 |
| Cardiovascular disease | 2.75 (1.57 – 4.80) | 0.021 |

95%CI: 95% confidence interval

We also have modified the results in the abstract (page 2, lines 40-41), and in the results section (page 12, lines 189-190)

Hence this result implies that patients should consult as early as possible in the acute phase and that is necessary to adjust for this delay to present the independent risk of other predictive factors.

Stratifying on delay, severe bleedings were the only severity component to be significantly associated with a consultation time greater than 2 days after onset. This is consistent with the literature which reports that delay in diagnosis is associated with severity including severe bleedings, complications, and mortality of dengue :

- Mallhi TH, Khan AH, Adnan AS, Sarriff A, Khan YH, Jummaat F. Clinico-laboratory spectrum of dengue viral infection and risk factors associated with dengue hemorrhagic fever: a retrospective study. BMC Infect Dis. 2015 Sep 30;15:399. doi: 10.1186/s12879-015-1141-3. PMID: 26423145; PMCID: PMC4590689.
- Chowell G, Diaz-Duenas P, Chowell D, et al. Clinical diagnosis delays and epidemiology of dengue fever during the 2002 outbreak in Colima, Mexico. Dengue Bull. 2007;31:26–35.
- Nguyen Thi KT, Nguyen Ngoc AT, Khau MT, Nguyen TT, Luong CQ. Epidemiology analysis of deaths associated with dengue hemorrhagic fever in Southern Viet Nam in 1999–2000. Dengue Bull. 2001;25:28–32.
- World Health Organization. Global strategy for dengue prevention and control 2012-2020. World Health Organization; 2012. Available: <https://apps.who.int/iris/handle/10665/75303> (*already cited in the manuscript*)

To supplement the conclusions, we added the sentence (page 15, lines 263-265): “Besides a history of cardiovascular disease and Western European origin that were associated with SD in our setting, the time to diagnosis and management of dengue is crucial and rehydration appears to play a central role in preventing progression to SD.”

We then changed in the conclusions of the abstract (page 2, lines 45-46): “It suggests that cardiovascular disease, Western European origin, and delay in diagnosis and management are risk factors associated with SD fever, and that restoration of blood volume and correction of dehydration must be performed early to be effective

Comment: My suggestion is to discuss the predictors and their potential interactions (based on other international references). Once they do that the paper is good to accept. I think this paper qualify for Minor revision.

Thus we discussed these results in the discussion section and added these references (page 14, lines 228-232):” Indeed, delay in diagnosis and management was also associated with SD. Severe bleedings were the only severity component to be significantly associated with a consultation time greater than 2 days after onset. Although the date of illness onset is declarative, this is consistent with the literature which reports that delay in diagnosis is associated with severity including severe bleedings, complications, and mortality of dengue [6,25-27].”

We hope you will find our revised version acceptable for publication in *PLOS Neglected Tropical Diseases* and look forward to hearing from you.

Yours sincerely,

Dr Olivier MAILLARD, PharmD, MSPH

Dr Antoine BERTOLOTTI, MD, PhD
